# An open-source high-speed infrared videography database to study the principles of active sensing in freely navigating rodents

**DOI:** 10.1093/gigascience/giy134

**Published:** 2018-11-10

**Authors:** Alireza Azarfar, Yiping Zhang, Artoghrul Alishbayli, Stéphanie Miceli, Lara Kepser, Daan van der Wielen, Mike van de Moosdijk, Judith Homberg, Dirk Schubert, Rémi Proville, Tansu Celikel

**Affiliations:** 1 Department of Neurophysiology, Donders Institute for Brain, Cognition, and Behaviour, Radboud University, Heyendaalseweg 135, Nijmegen, 6525 HJ The Netherlands; 2 Department of Cognitive Neuroscience, Donders Institute for Brain, Cognition, and Behaviour, Radboud University Medical School, Kapittelweg 29, Nijmegen, 6525 EN The Netherlands

**Keywords:** mystacial vibrissae, object localization, goal-directed behavior, mouse, rat, sensorimotor computation, whisking

## Abstract

**Background:**

Active sensing is crucial for navigation. It is characterized by self-generated motor action controlling the accessibility and processing of sensory information. In rodents, active sensing is commonly studied in the whisker system. As rats and mice modulate their whisking contextually, they employ frequency and amplitude modulation. Understanding the development, mechanisms, and plasticity of adaptive motor control will require precise behavioral measurements of whisker position.

**Findings:**

Advances in high-speed videography and analytical methods now permit collection and systematic analysis of large datasets. Here, we provide 6,642 videos as freely moving juvenile (third to fourth postnatal week) and adult rodents explore a stationary object on the gap-crossing task. The dataset includes sensory exploration with single- or multi-whiskers in wild-type animals, serotonin transporter knockout rats, rats received pharmacological intervention targeting serotonergic signaling. The dataset includes varying background illumination conditions and signal-to-noise ratios (SNRs), ranging from homogenous/high contrast to non-homogenous/low contrast. A subset of videos has been whisker and nose tracked and are provided as reference for image processing algorithms.

**Conclusions:**

The recorded behavioral data can be directly used to study development of sensorimotor computation, top-down mechanisms that control sensory navigation and whisker position, and cross-species comparison of active sensing. It could also help to address contextual modulation of active sensing during touch-induced whisking in head-fixed vs freely behaving animals. Finally, it provides the necessary data for machine learning approaches for automated analysis of sensory and motion parameters across a wide variety of signal-to-noise ratios with accompanying human observer-determined ground-truth.

## Data Description

### Context

Whiskers, or mystacial vibrissae, are sensory hairs that are densely organized as a grid on the snout. Rats and mice actively move their whiskers in an oscillatory motion to explore their environment as they integrate sensory information spatiotemporally across whiskers and whisk cycles [1–5]. The motor control of whisker position is a result of sensorimotor computation where sensory information collected during the last ∼3 whisk cycles is used to plan the whisker motion for the subsequent whisk cycle [6]. Although animals can perceive passive touch before the onset of whisking [7], it is not known when and where in the brain the sensorimotor computation for adaptive motor control for whisker position emerges. Moreover, the mechanisms responsible for the development and plasticity of sensorimotor computation are largely unknown. Because sensorimotor integration is contextually regulated [8–12], altered by the change in neuronal excitability along the sensorimotor circuits [13] and based on experience and the current state of the sensory organs [1], identification of the principles of sensorimotor computation will require large-scale behavioral experiments where sensory input on whiskers and motor control of whisker position are studied at high spatiotemporal resolution. Here, we introduce the first iteration of such a dataset as freely moving rodents locate a tactile target under infrared light. The dataset includes independent variables of species (rat vs mouse), developmental age (juvenile vs adult, i.e., 3–5 postnatal weeks and >6 weeks, respectively), sensory deprivation (single vs multi-whisker,) and genetic background (i.e., serotonin transporter [SERT] knock-down vs control]; see below). The database might serve researchers across a broad range of disciplines, including cellular, behavioral, systems, cognitive neuroscience, ethology, biomimetics, robotics, artificial intelligence, computer vision, and active sensing communities, to study and model the principles of active sensing.

### Animals

All experiments have been performed according to the Dutch law concerning animal welfare and the guidelines for the care and use of laboratory animals upon institutional ethical committee approval. All efforts have been made to minimize animal suffering and discomfort, and all precautions were taken to reduce the number of animals used.

The experiments were performed on 38 male rats and 10 male mice. Rats were either genetically engineered or pharmacologically treated to alter serotonergic neurotransmission, a neuromodulatory neurotransmitter that contributes to motor control [14], stimulus encoding in the barrel cortex [15], and is believed to modulate development and maturation of sensorimotor circuits [16]. Experiments in rats also included corresponding wild-type and vehicle injection controls. Mice were on the C57Bl6 background (B6;129P2-Pvalbtm1(cre)Arbr/J, the Jackson Laboratory, RRID:MGI:5315557). Parvalbumin neurons in this line express Cre-recombinase, but the mice were otherwise not genetically or pharmacologically altered. The founder line was outcrossed to C57Bl6 for 20+ generations before the start of experiments. All mice were studied when between 2 and 4 months of age.

SERT knockout (KO) rats (Slc6a41Hubr) were generated on a Wistar background by N-ethyl-N-nitrosurea-induced mutagenesis as described before [17]. Experimental animals were derived from heterozygous 5-HT transporter KO (5HTT^−/−^) rats that were outcrossed for 12+ generations with wild-type Wistar rats obtained from Harlan Laboratories (Horst, The Netherlands). Ear punches were taken at the age of 21 days after weaning for genotyping; 5HTT^−/−^ and 5HTT^+/+^ rats were randomly assigned to SERT KO (N = 14 rats) and wild-type groups (N = 14 rats), respectively.

The 5-HT transporter deletion alters neural function starting from embryonic brain development [17]. Thus, in a second group of rats, we interfered with the serotonergic system after birth and only transiently when serotonergic innervations appear in the barrel cortex [9]. Fluoxetine hydrochloride (10 mg/kg/day, Sigma Aldrich), a selective serotonin reuptake inhibitor, was dissolved in water and administered orally. Age-matched dams in a separate cage received tap water and were considered as vehicle controls. The fluoxetine administration started after birth (P1) and continued for 7 days, corresponding to the period of postnatal development critical for the maturation of thalamocortical projections [18]. The pups of all groups (fluoxetine, N = 5; vehicle, N = 5) were kept together with their mothers until weaning.

### Animal handling and behavioral observations

**Figure 1:**
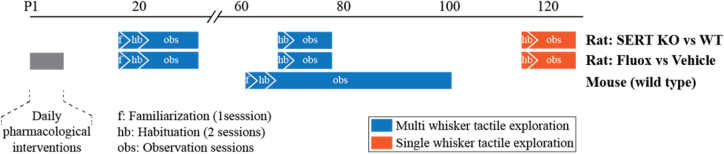
The timeline of experiments and handling. See main text for details.

Animal behavior was studied as they located (or attempted to locate) a tactile target under infrared light between postnatal days (P) 21-P30, i.e., as juveniles, and/or after they reached sexual maturity (Fig. 1). Animal-handling protocols were similar to those employed previously [1, 6, 8, 13]. Experiments started with a familiarization session (20 minutes per animal) where P18 pup (in rats) or adult mouse subjects were introduced to the experimenter and the experimental room the first time. Habituation to the setup consisted of two 20-minute sessions under no visible light but with white noise. The training sessions (N = 10/rat; N = 30/mouse) lasted 30 minutes (or 30 successful trials) in which the gap distance (see below) was randomly drawn from a Gaussian distribution. With increasing number of sessions, the mean of the distribution was increased and variance reduced, adapting each animal's individual learning curve to ensure animals preferentially use their whiskers for target localization in the majority of the trials. The setup was cleaned with ethanol between sessions.

One day before the sessions that required animals to perform the task with a restricted set of whiskers, animals were anesthetized using isoflurane. Half of the animals received whisker plucking sparing a single (C2) whisker or single (C) row bilaterally; the other half received “sham plucking” during which they were handled similarly to the whisker-deprived animals; however, their whiskers were left unplucked. Whisker regrowth was assessed every day; if needed, whisker plucking was repeated.

### The behavioral paradigm: tactile object localization

We observed animals, under infrared light, as they shuttled between two elevated platforms with a variable gap-distance in between them. The animals were not food deprived, nor did they receive any reward for successful task execution. In this, so-called spontaneous gap-crossing task [1, 6, 8, 13], the distance between the platforms is varied to enable observation of whisker-dependent tactile object localization. In our training protocols, the gap-distance was randomly selected from a normal distribution whose mean increases and variance reduces with repeated training (i.e., increased number of training sessions) as described elsewhere [15]. Catch trials, where the target platform is positioned just outside of the animal's reach, were randomly introduced (∼15% of successful trials) to ensure that the task execution required tactile exploration and was not a result of expectation and sensorimotor habit formation.

### Experimental setup and data acquisition

**Figure 2:**
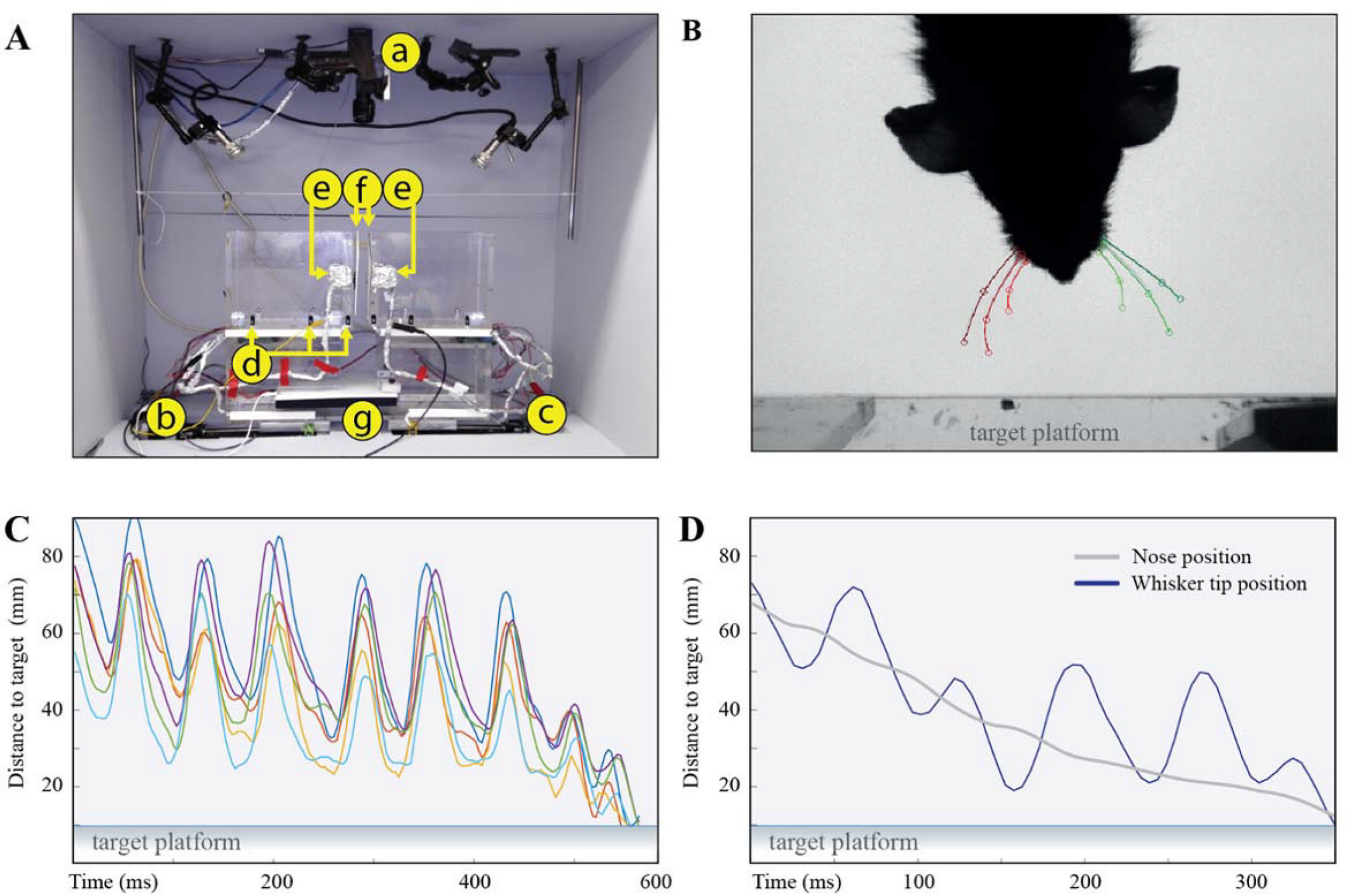
Experimental setup and sample behavioral data. **(A)** The experimental setup is installed in a sound-attenuated chamber. Three linear actuators (a-c) mobilize a high-speed camera and tactile targets. Infrared motion sensors (d; 3x/platform) provide positional information about the animal and gate all actuators. Servo motors (e) installed at the ends of the platforms by the gap mobilize polyvinyl chloride panels (f) that act as gates. Gates are closed between trials and during tactile target motion. A custom-made infrared (890 nm) panel provides background illumination for the video recordings. **(B)** A sample still image with human observers’ ground truth data about whisker positions are overlaid. Images were acquired at either 480 fps with a resolution of 512 × 640 (110 microm/pixel) using a PointGrey Flea3 (FLIR, Germany) camera (in mice) or at 220 fps (240 × 320 pixels; 625 microm/pixel) using an AVT Pike (Allied Vision, Germany) camera (in rats). **(C)** Whisker tip position for six whiskers as a rat located the target. Each color corresponds to one whisker. **(D)** Similar to (C) but for single whisker along with the corresponding trace of nose position.

The experimental setup consists of two elevated platforms and a high-speed camera that are mobilized by linear actuators (Fig. 2A). The animal positions on the platforms are tracked using motion sensors. Motion sensors also provide real-time feedback for robotic actions including closure of doors, limiting the animal's access to the gap, gating the sequence that controls the position of tactile targets, triggering the streaming of high-speed videography data to disk, repositioning the camera to ensure optimal field of view independent from the target location, and, if required, delivery of the reward.

Each session starts with the experimenter positioning the animal on one of the two platforms. The task of the animal in any given trial is to locate the other platform, if it is within tactile reach. The success on the task is defined as the animal traveling between the two far ends of the platforms, as assessed by motion sensors in real time. If an animal starts and returns to the same starting position without interrupting the middle motion sensor on the other platform, the trial is classified as a failure. Animals are allowed to visit the gap as many times as they require before making a decision on whether or not to gap-cross. Upon decision, the door attached to the only access point of the platform that the animal is located upon is closed, and the target platform is positioned in its new position as described above.

Animals’ sensorimotor behavior as they attempt to locate the target is recorded using a high-speed camera. The camera is mobilized using a linear actuator to ensure a comparable field of view across trials. An infrared backlight is positioned below the gap to provide the necessary contrast for imaging (Fig. 2B).

The videography data can be used to track body and whisker position in high spatiotemporal resolution. To provide the ground-truth data for future machine learning approaches for whisker tracking, three human observers tracked whisker and nose position in a nonoverlapping subset of videos (>150 tracked frames/video). Corresponding raw data are provided in .mat (MATLAB) format; see Fig. 2C and D for sample traces; see Supplementary Table S1 for a list of files that include ground-truth tracking data. If animals made multiple attempts to locate the target, which is common especially during the early phases of object localization training [1], the human observers were instructed to focus on the last epoch of exploration.

### Data format and online database organization

All video files are stored as four-dimensional matrices in .mat files as well as .mp4 files for streamlined navigation in standard browsers. The .mat formatted data can be visualized using the “implay” function in the Image Processing Toolbox or using the standard “movie” function in MATLAB. Movies can be converted to other formats using built-in functions “movie2avi” or “videowriter.” The videos can be manually or automatically segmented using open-source software (e.g., [19–21]

**Figure 3:**
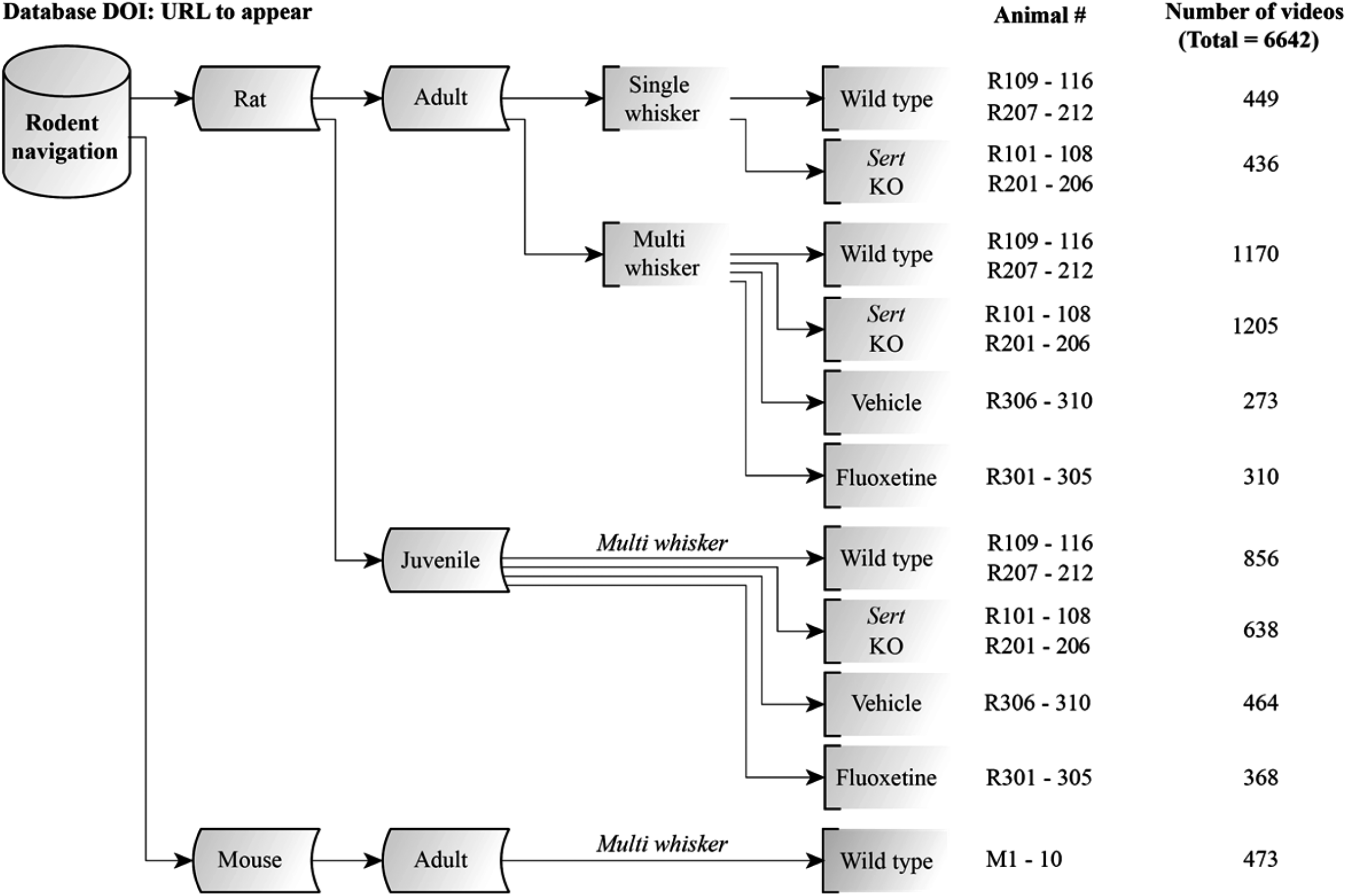
Organization of the dataset. See main text for details.

The data are available online [22]. The hierarchy in the data organization is shown in Fig. 3 and includes, in descending order, species (rat vs mouse), age (juvenile vs adult), sensory exploration with single or multiple whiskers (e.g., single row or all whiskers intact), and transgenic methods of intervention with serotonergic signaling along with corresponding controls. A tabulated Excel document (Supplementary Table S1) provides the metadata about the experimental details, including date of experiment, session and trial numbers, gap-distance, trial outcome (success vs failure), and whether the video is human clicked.

### Data Validation and Quality Control

Video acquisition was initiated when the animal triggered a motion sensor located at the start of the gap while standing at the edge of the home platform. Despite having started the gap-exploration, animals often opt to abort the search for the target platform before advancing toward the target. Therefore, all videos were screened individually using a custom-written software in MATLAB; only those videos where animals successfully located the target platform were included in the database.

Whiskers in a proportion of the database (619/6642 videos) were manually tracked by human observers using a custom-written interface in MATLAB [19]. The observers monitored a nonoverlapping set of videos. The number of frames tracked varied across videos as the duration of exploration is not constant across trials, but was >150 frames/video, six whiskers/frame.

### Application scenarios

This database will help to address numerous fundamental questions in systems neuroscience, including but not limited to, development of sensorimotor computation, top-down mechanisms that control sensory navigation and whisker position, and cross-species comparison of active sensing. By comparing the sensorimotor exploration across wild-type juvenile and adult animals, one could address how adaptive control of body and whisker position develop. Because adaptive motor control of whiskers is likely to be an outcome of a vector computation that ensures spatial constancy despite the coupled changes in the body [2], developmental changes in body positional control with respect to whisking might unravel the sequential development of motor control. Repeating the same analysis across SERT KO, Fluoxetine, and the corresponding control animals would help to address the role of serotonin in shaping motor development and consequences of altered serotonergic signaling in sensorimotor control in adulthood. Finally, by comparing the sensorimotor exploration between the multi-whiskered rats and mice, one could address cross-species differences in adaptive motor control during object localization.

The data provided could serve the ongoing machine learning efforts that will ultimately allow automated segmentation of whiskers in near real time, i.e., in temporal resolution shorter than the duration of a whisk cycle. To ensure the usability of the database as a training set, we have included ground-truth data from a subset of video recordings. Understanding the principles of active sensing in biological systems might help to instruct adaptive solutions for artificial systems to adapt sensory navigation to the ever-changing motor demands of the navigating agent.

### Limitations

Freely behaving animal experiments are often burdened by high dimensionality and the associated sampling limitations. Even if animals execute behavior in a constrained environment, e.g., exploring a stationary target while standing on an elevated platform, as in the behavioral experiments described herein, animals could change their approach angle, kinematics of whisking, duration of exploration, number of whiskers used to sample the target, head angle, and head elevation, among other variables across different trials. Previous studies quantifying the sensory, motor, and perceptual behavior during whisker-based object localization showed that both rats and mice perform spontaneous gap-crossing in a stereotypical manner and that ∼100 trials (10 trials/animal) is sufficient to gather reproducible statistics of sensory and motor behaviors [1, 6, 8, 13, 15, 22]. Thus, the current dataset with 6,642 independent observations across 11 independent conditions (including species, age, genetics, pharmacological and sensory deprivation interventions) should provide sufficient sampling to address fundamental questions outlined in the previous section. However, it is important to note that the dataset does not include data from female animals.

## Availability of source code and requirements

Project name: MATLAB Whisker Tracker

Project home page: https://github.com/DepartmentofNeurophysiology/Matlab-Whisker-Tracker

Operating system(s): Platform independent

Programming language: MATLAB

Other requirements: MATLAB 2017a or higher

License: GNU GPL

RRID:SCR_016538

## Availability of supporting data

Snapshots of the database and code, including further supporting data, are available in the *GigaScience* repository, GigaDB [23].

## Additional files

Azarfar_Metadata_Supplemental Table1.xlsx

## Abbreviations

KO: knockout; SERT: serotonin transporter.

## Competing interests

The authors declare that they have no competing interests.

## Funding

This work was supported by the European Commission (Horizon2020, nr. 660328), European Regional Development Fund (MIND, nr. 122035), and the Netherlands Organisation for Scientific Research (NWO-ALW Open Competition, nr. 824.14.022).

## Author contributions

Investigation: A.A., Y.Z., A.r.A.,S.M., L.K., D.v.d.W., M.v.d.M.. Formal analysis and data curation: A.A.. Software: A.A., R.P., T.C.. Validation: A.A., Y.Z., A.r.A., S.M., L.K., D.v.d.W., M.v.d.M.. Resources: J.H., D.S., T.C.. Visualization and Writing (original draft preparation): A.A., T.C.. Writing (review and editing):A.A., Y.Z., A.r.A., S.M., L.K., D.v.d.W., M.v.d.M., J.H., D.S., R.P., T.C.. Conceptualization, Supervision, Project Administration, Methodology, Funding Acquisition: T.C.

**Table:** 

Contributor Role	Author
**Conceptualization**	T.C.
**Supervision**	T.C.
**Project Administration**	T.C.
**Investigation**	A.A., Y.Z., A.r.A.,S.M., L.K., D.v.d.W., M.v.d.M.
**Formal Analysis**	AA
**Software**	AA, R.P., T.C.
**Methodology**	T.C.
**Validation**	A.A., Y.Z., A.r.A., S.M., L.K., D.v.d.W., M.v.d.M.
**Data Curation**	A.A.
**Resources**	TC, J.H., D.S.
**Funding Acquisition**	T.C.
**Writing—Original Draft Preparation**	A.A., T.C.
**Writing—Review & Editing**	A.A., Y.Z., A.r.A., S.M., L.K., D.v.d.W., M.v.d.M., J.H., D.S., R.P., T.C.
**Visualization**	A.A., T.C.

## Supplementary Material

GIGA-D-18-00333_Original_Submission.pdf

GIGA-D-18-00333_Revision_1.pdf

Response_to_Reviewer_Comments_Original_Submission.pdf

Reviewer_1_Report_(Original_Submission).pdf

Supplement File
