# Supplementary material for: An open-source high-speed infrared videography database to study the principles of active sensing in freely navigating rodents
Source: Gigascience. 2018 Nov 10;7(12):giy134. doi: 10.1093/gigascience/giy134 (PMC6283211; doi:10.1093/gigascience/giy134)
Supplement: GIGA-D-18-00333_Original_Submission.pdf [file giy134_giga-d-18-00333_original_submission.pdf]

GigaScience

An open-source high-speed infrared videography database to study the principles of active sensing in freely navigating rodents

--Manuscript Draft--

**Manuscript Number:**	GIGA-D-18-00333
**Full Title:**	An open-source high-speed infrared videography database to study the principles of active sensing in freely navigating rodents
**Article Type:**	Data Note
**Funding Information:**	H2020 European Research Council (660328) — Dr. Tansu Celikel<br>Interreg (122035) — Dr. Tansu Celikel<br>Nederlandse Organisatie voor Wetenschappelijk Onderzoek (824.14.022) — Dr. Tansu Celikel
**Abstract:**	Background: Active sensing is crucial for navigation. It is characterized by self-generated motor action controlling the accessibility and processing of sensory information. In rodents, active sensing is commonly studied in the whisker system. As rats and mice modulate their whisking contextually, they employ frequency and amplitude modulation. Understanding the development, mechanisms and plasticity of adaptive motor control will require precise behavioral measurements of whisker position.<br>Findings: Advances in high-speed videography and analytical methods now permit collection and systematic analysis of large datasets. Here we provide 6642 videos as freely moving juvenile (3rd-4th postnatal week) and adult rodents explore a stationary object on the gap-crossing task. The dataset includes sensory exploration with single- or multi-whiskers in wild-type animals, serotonin transporter knock-out rats, rats received pharmacological intervention targeting serotonergic signaling. The dataset includes varying background illumination conditions and signal-to-noise ratios (SNRs), ranging from homogenous/high contrast to non-homogenous/low-contrast. A subset of videos has been whisker and nose tracked, and are provided as reference for image processing algorithms.<br>Conclusions: The recorded behavioral data can be directly used to study (1) development of sensorimotor computation, (2) top-down mechanisms that control sensory navigation and whisker position, (3) cross-species comparison of active sensing. It could also help to address contextual modulation of active sensing during touch induced whisking in head-fixed versus freely behaving animals. And finally, it provides the necessary data for machine learning approaches for automated analysis of sensory and motion parameters across a wide variety of SNRs with accompanying human observer determined ground-truth.
**Corresponding Author:**	Tansu Celikel, PhD<br>Donders Institute for Brain, Cognition and Behaviour<br>NETHERLANDS
**Corresponding Author Secondary Information:**	
**Corresponding Author's Institution:**	Donders Institute for Brain, Cognition and Behaviour
**Corresponding Author's Secondary Institution:**	
**First Author:**	Alireza Azarfar
**First Author Secondary Information:**	
**Order of Authors:**	Alireza Azarfar<br>Yiping Zhang<br>Artoghrul Alishbayli

Powered by Editorial Manager® and ProduXion Manager® from Aries Systems Corporation

	Stéphanie Miceli
	Lara Kepser
	Daan van der Wielen
	Mike van de Moosdijk
	Judith Homberg
	Dirk Schubert
	Rémi Proville
	Tansu Celikel, PhD
**Order of Authors Secondary Information:**	
**Additional Information:**	
**Question**	**Response**
Are you submitting this manuscript to a special series or article collection?	No
**Experimental design and statistics**<br><br>Full details of the experimental design and statistical methods used should be given in the Methods section, as detailed in our Minimum Standards Reporting Checklist. Information essential to interpreting the data presented should be made available in the figure legends.<br><br>Have you included all the information requested in your manuscript?	Yes
**Resources**<br><br>A description of all resources used, including antibodies, cell lines, animals and software tools, with enough information to allow them to be uniquely identified, should be included in the Methods section. Authors are strongly encouraged to cite Research Resource Identifiers (RRIDs) for antibodies, model organisms and tools, where possible.<br><br>Have you included the information requested as detailed in our Minimum Standards Reporting Checklist?	Yes
**Availability of data and materials**	Yes

All datasets and code on which the conclusions of the paper rely must be either included in your submission or deposited in publicly available repositories (where available and ethically appropriate), referencing such data using a unique identifier in the references and in the “Availability of Data and Materials” section of your manuscript.<br><br>Have you have met the above requirement as detailed in our Minimum Standards Reporting Checklist?	

A Datanote submission to GigaScience

An open-source high-speed infrared videography database to study the principles of active sensing in freely navigating rodents

Alireza Azarfar[1], Yiping Zhang[1], Artoghrul Alishbayli[1], Stéphanie Miceli[2, CA], Lara Kepser[2], Daan van der Wielen[1], Mike van de Moosdijk[1], Judith Homberg[2], Dirk Schubert[2,1], Rémi Proville[1], Tansu Celikel[1*]

(1) Department of Neurophysiology, Donders Institute for Brain, Cognition, and Behaviour, Radboud University, Nijmegen - the Netherlands. (2) Department of Cognitive Neuroscience, Donders Institute for Brain, Cognition, and Behaviour, Radboud University Medical School, Nijmegen - the Netherlands. [CA] Current address: Center of Advanced European Studies and Research, Bonn, Germany

E-mail addresses: a.azarfar@neurophysiology.nl, y.zhang@neurophysiology.nl, a.alishbayli@neurophysiology.nl, stephanie.miceli@caesar.de, larakepser11@gmail.com, d.vanderwielen@neurophysiology.nl, m.vandemoosdijk@neurophysiology.nl, judith.homberg@radboudumc.nl, d.schubert@donders.ru.nl, r.proville@neurophysiology.nl, t.celikel@neurophysiology.nl

*Corresponding author: Tansu Celikel

3 Figures, 1 Supplemental Table, 19 References

Keywords: Mystacial vibrissae, object localization, goal-directed behavior, mouse, rat, sensorimotor computation, whisking

Funding:

This work was supported by the European Commission (Horizon2020, nr. 660328), European Regional Development Fund (MIND, nr. 122035) and the Netherlands Organisation for Scientific Research (NWO-ALW Open Competition, nr. 824.14.022).

Abstract

Background: Active sensing is crucial for navigation. It is characterized by self-generated motor action controlling the accessibility and processing of sensory information. In rodents, active sensing is commonly studied in the whisker system. As rats and mice modulate their whisking contextually, they employ frequency and amplitude modulation. Understanding the development, mechanisms and plasticity of adaptive motor control will require precise behavioral measurements of whisker position.

Findings: Advances in high-speed videography and analytical methods now permit collection and systematic analysis of large datasets. Here we provide 6642 videos as freely moving juvenile (3rd-4th postnatal week) and adult rodents explore a stationary object on the gap-crossing task. The dataset includes sensory exploration with single- or multi-whiskers in wild-type animals, serotonin transporter knock-out rats, rats received pharmacological intervention targeting serotonergic signaling. The dataset includes varying background illumination conditions and signal-to-noise ratios (SNRs), ranging from homogenous/high contrast to non-homogenous/low-contrast. A subset of videos has been whisker and nose tracked, and are provided as reference for image processing algorithms.

Conclusions: The recorded behavioral data can be directly used to study (1) development of sensorimotor computation, (2) top-down mechanisms that control sensory navigation and whisker position, (3) cross-species comparison of active sensing. It could also help to address contextual modulation of active sensing during touch induced whisking in head-fixed versus freely behaving animals. And finally, it provides the necessary data for machine learning approaches for automated analysis of sensory and motion parameters across a wide variety of SNRs with accompanying human observer determined ground-truth.

Data description

Context

Whiskers, or mystacial vibrissae, are sensory hairs that are densely organized as a grid on the snout. Rats and mice actively move their whiskers in an oscillatory motion to explore their environment as they integrate sensory information spatiotemporally across whiskers and whisk cycles [1–5]. The motor control of whisker position is a result of sensorimotor computation where sensory information collected during the last ~3 whisk cycles is used to plan the whisker motion for the subsequent whisk cycle [6]. Although animals can perceive passive touch before the onset of whisking [7], it is not known when and where in the brain the sensorimotor computation for adaptive motor control for whisker position emerge. Moreover, the mechanisms responsible for the development and plasticity of sensorimotor computation are largely unknown. Because sensorimotor integration is contextually regulated [8–12], altered by the change in neuronal excitability along the sensorimotor circuits [13] and based on experience and the current state of the sensory organs [1], identification of the principles of sensorimotor computation will require large scale behavioral experiments where sensory input on whiskers and motor control of whisker position are studied at high spatiotemporal resolution. Here we introduce the first iteration of such a dataset as freely moving rodents locate a tactile target under infrared light. The dataset includes independent variables of species (rat vs mouse), developmental age (juvenile vs adult, i.e. 3-5 postnatal weeks and >6 weeks, respectively), sensory deprivation (single vs multi whisker) and genetic background (i.e. SERT knock-down vs control, see below). The database might serve researchers across a broad range of disciplines, including cellular, behavioral, systems, cognitive neuroscience, and ethology, biomimetics,

robotics, artificial intelligence, computer vision and active sensing communities, to study and model the principles of active sensing.

Animals

All experiments have been performed according to the Dutch law concerning animal welfare and the guidelines for the care and use of laboratory animals upon institutional ethical committee approval. All efforts have been made to minimize animal suffering and discomfort, and all precautions were taken to reduce the number of animals used.

The experiments were performed on 38 male rats and 10 male mice. Rats were either genetically engineered or pharmacologically treated to alter serotonergic neurotransmission, a neuromodulatory neurotransmitter that contributes to motor control [14], stimulus encoding in the barrel cortex [15], and is believed to modulate development and maturation of sensorimotor circuits [16]. Experiments in rats also included corresponding wild type and vehicle injection controls. Mice were on the C57Bl6 background (B6;129P2-Pvalbtm1(cre)Arbr/J, The Jackson Laboratory, RRID:MGI:5315557). Parvalbumin neurons in this line express Cre-recombinase but the mice were otherwise not genetically or pharmacologically altered. The founder line was outcrossed to C57Bl6 for 20+ generations before the start of experiments. All mice were studied between 2-4 months of age.

Serotonin transporter knockout rats (Slc6a41Hubr) were generated on a Wistar background by N-ethyl-N-nitrosurea (ENU)-induced mutagenesis as described before [17]. Experimental

animals were derived from heterozygous 5-HT transporter knockout (5HTT$^{-/-}$) rats that were outcrossed for 12+ generations with wild-type Wistar rats obtained from Harlan Laboratories (Horst, The Netherlands). Ear punches were taken at the age of 21 days after weaning for genotyping and 5HTT$^{-/-}$ and 5HTT$^{+/+}$ rats were randomly assigned to SERT KO (N=14 rats) and WT groups (N=14 rats), respectively.

5-HT transporter deletion alters neural function starting from embryonic brain development [17]. Thus, in a second group of rats, we interfered with the serotonergic system after birth, and only transiently when serotonergic innervations appear in the barrel cortex [9]. Fluoxetine hydrochloride (10 mg/kg/day, Sigma Aldrich), a selective serotonin reuptake inhibitor, was dissolved in water and administered orally. Age matched dams in a separate cage received tap water and were considered as Vehicle controls. The fluoxetine administration started after birth (P1) and continued for 7 days, corresponding to the period of postnatal development critical for the maturation of thalamocortical projections [18]. The pups of all groups (Fluoxetine, N=5; Vehicle, N=5) were kept together with their mothers until weaning.

Animal handling and behavioral observations

Animal behavior was studied as they located (or attempted to locate) a tactile target under infrared light between postnatal days (P) 21-P30, i.e. as juveniles, and/or after they reached sexual maturity (Figure 1). Animal handling protocols were similar to those employed previously [1, 6, 8, 13]. Experiments started with a familiarization session (20 min/animal) where P18 pup (in rats) or adult mouse subjects were introduced to the experimenter and the experimental room

the first time. Habituation to the set-up consisted of two 20 minute sessions under no visible light but with white noise. The training sessions (N=10/rat; N=30/mouse) lasted 30 minutes (or 30 successful trials) in which the gap distance (see below) was randomly drawn from a Gaussian distribution. With increasing number of sessions, the mean of the distribution was increased and variance reduced, adapting each animal's individual learning curve, to ensure animals preferentially use their whiskers for target localization in majority of the trials. The set-up was cleaned with ethanol between sessions.

One day before the sessions that required animals to perform the task with a restricted set of whiskers, animals were anesthetized using isoflurane. Half of the animals received whisker plucking sparing a single (C2) whisker or single (C) row bilaterally; the other half received "sham plucking" during which they were handled similarly to the whisker deprived animals, however their whiskers were left unplucked. Whisker regrowth was assessed every day, and if needed whisker plucking was repeated.

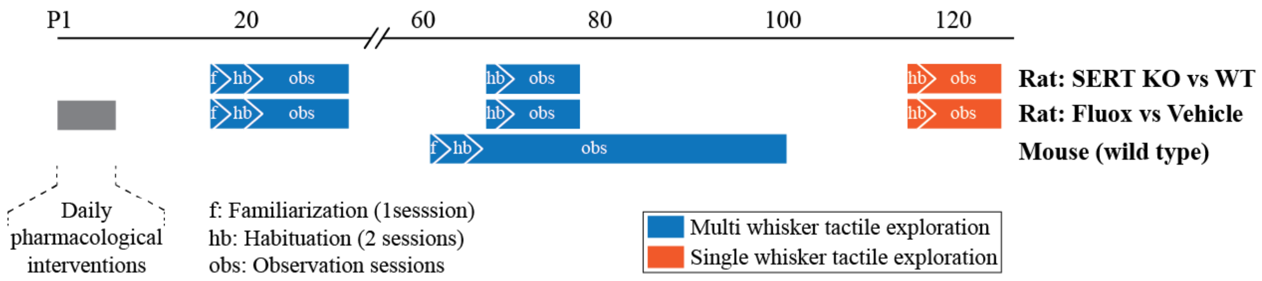

Figure 1. The timeline of experiments and handling. See main text for details.

The behavioral paradigm: Tactile object localization

We observed animals, under infrared light, as they shuttled between two elevated platforms with a variable gap-distance in between them. The animals were not food deprived; neither did they receive any reward for successful task execution. In this, so called spontaneous gap-crossing task [1, 6, 8, 13], the distance between the platforms is varied to enable observation of whisker dependent tactile object localization. In our training protocols, the gap-distance was randomly selected from a normal distribution whose mean increases and variance reduces with repeated training (i.e. increased number of training sessions) as described before [15]. Catch trials, where the target platform is positioned just outside of the animal's reach, were randomly introduced (~15% of successful trials) to ensure that the task execution required tactile exploration and was not a result of expectation and sensorimotor habit formation.

The experimental set-up and data acquisition

The experimental set-up consists of two elevated platforms and a high-speed camera that are mobilized by linear actuators (Figure 2A). The animal position on the platforms are tracked using motion sensors. Motion sensors also provide real-time feedback for robotic actions including closure of doors, limiting the animal's access to the gap, gating the sequence that control the position of tactile targets, triggering the streaming of high-speed videography data to disk, repositioning the camera to ensure optimal field-of-view independent from the target location, and, if required, delivery of the reward.

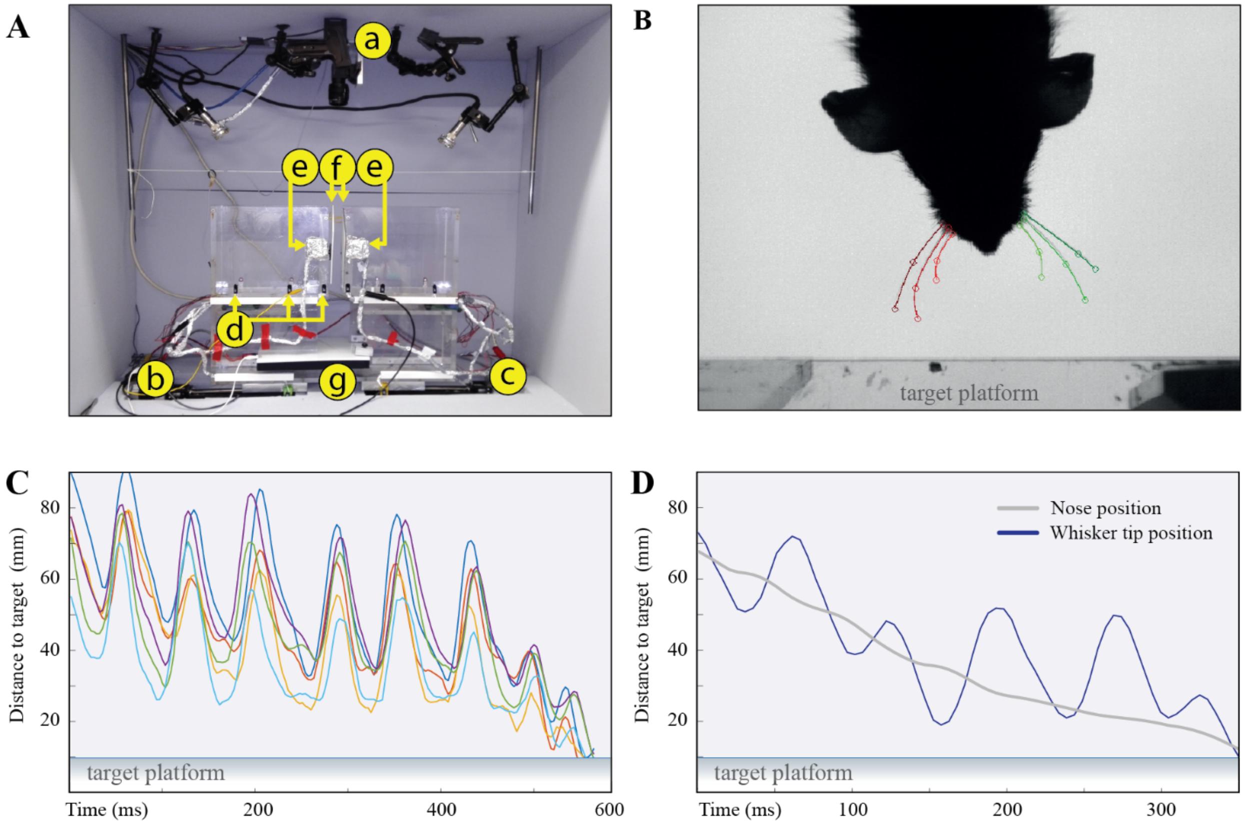

Figure 2. Experimental set-up and sample behavioral data. (A) The experimental set-up is installed in a sound attenuated chamber. Three linear actuators (a-c) mobilize a high-speed camera and tactile targets. Infrared motion sensors (d; 3x/platform) provide positional information about the animal and gate all actuators. Servo motors (e) installed at the ends of the platforms by the gap mobilize PVC panels (f) that act as gates. Gates are closed between trials and during tactile target motion. A custom made infrared (890nm) panel provide background illumination for the video recordings. **(B)** A sample still image with human observers' ground truth data about whisker positions are overlaid. Images were acquired at either 480fps with a resolution of 512x640 (110 microm/pixel) using a PointGrey Flea3 (FLIR, Germany) camera (in mice) or at 220fps (240x320 pixels; 625 microm/pixel) using an AVT Pike (Allied Vision, Germany) camera (in rats). **(C)** Whisker tip position for 6 whiskers as a rat located the target. Each color corresponds to one whisker. **(D)** Similar to C but for single whisker along with the corresponding trace of nose position.

Each session starts with the experimenter positioning the animal on one of the two platforms. The task of the animal in any given trial is to locate the other platform, if it is within tactile reach. The success on the task is defined as the animal traveling between the two far ends of the platforms, as assessed by motion sensors in real-time. If an animal starts and returns to the same starting position without interrupting the middle motion sensor on the other platform, the trial is classified as a failure. Animals are allowed to visit the gap as many time as they require before making a decision on whether or not to gap-cross. Upon decision, the door attached to the only access point of the platform that the animal is located upon is closed and the target platform is positioned in its new position as described above.

Animals' sensorimotor behavior as they attempt to locate the target is recorded using a high-speed camera. The camera is mobilized using a linear actuator to ensure comparable field of view across trials. An infrared backlight is positioned below the gap to provide the necessary contrast for imaging (Figure 2B).

The videography data can be used to track body and whisker position in high spatiotemporal resolution. To provide the ground-truth data for future machine learning approaches for whisker tracking, three human observers tracked whisker and nose position in a non-overlapping subset of videos (>150 tracked frames/video). Corresponding raw data are provided in .mat (MATLAB) format, see Figure 2C and 2D for sample traces, see Supplemental Table 1 for list of files that include ground-truth tracking data. If animals made multiple attempts to locate the target, which

is common especially during the early phases of object localization training (Celikel and Sakmann 2007), the human observers were instructed to focus on the last epoch of exploration.

Data format and online database organization

All video files are stored as 4D matrices in .mat files as well as .mp4 files for streamlined navigation in standard browsers. The .mat formatted data can be visualized using “implay” function in the Image Processing Toolbox or using the standard “movie” function in MATLAB. Movies can be converted to other formats using built in functions “movie2avi” or “videowriter”. The videos can be manually or automatically segmented using open source software (e.g. https://bitbucket.org/benglitz/controller-dnp/downloads/, https://github.com/AlexEMG/DeepLabCut, https://github.com/DepartmentofNeurophysiology/Matlab-Whisker-Tracker)

The data is available online (DOI to appear here). The hierarchy in the data organization is shown in Figure 3 and include, in descending order, species (rat vs mouse), age (juvenile vs adult), sensory exploration with single or multiple whiskers (e.g. single row or all whiskers intact) and transgenic, methods of intervention with serotonergic signaling along with corresponding controls. A tabulated excel document (Supplemental Table 1) provides the metadata about the experimental details including date of experiment, session and trial numbers, gap-distance, trial outcome (success vs failure), and whether the video is human clicked.

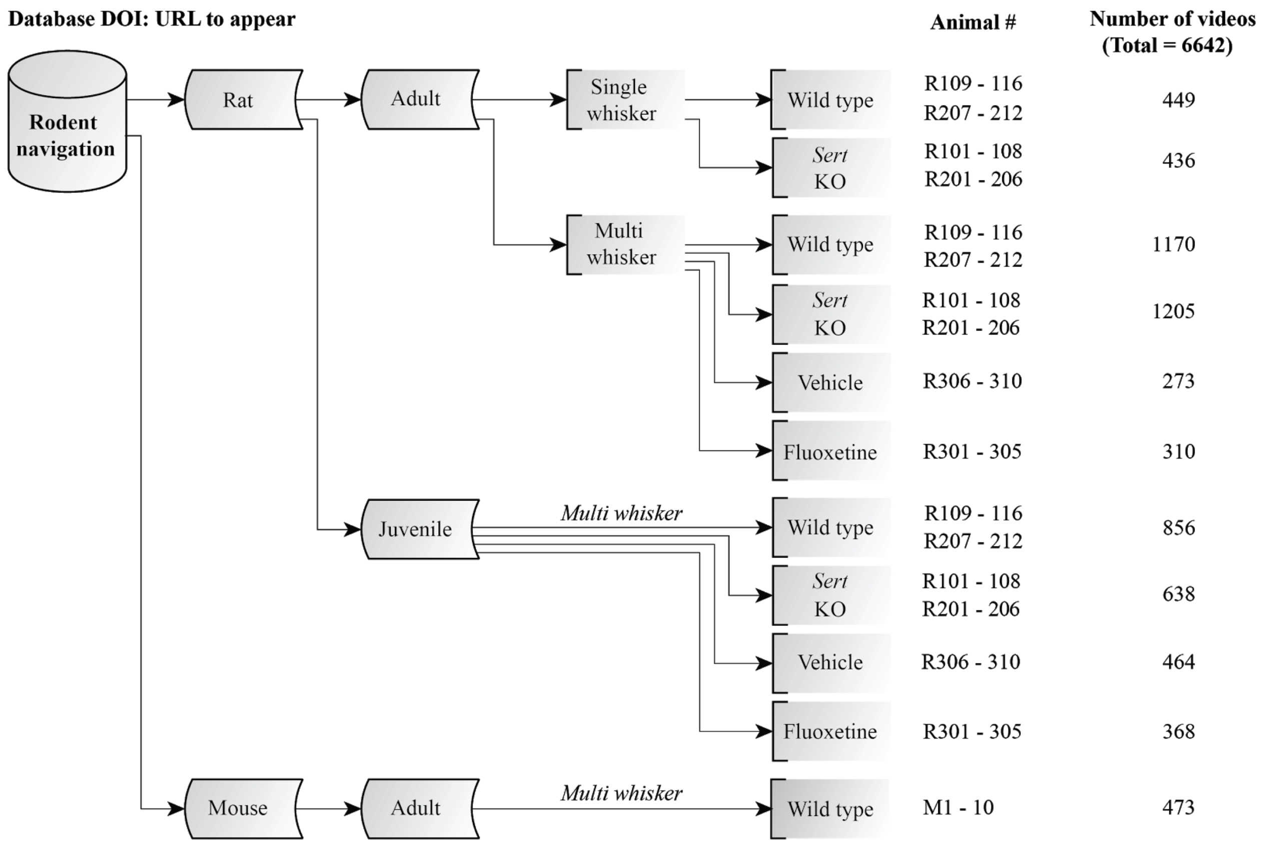

Figure 3. Organization of the dataset. See main text for details.

Application scenarios

This database will help to address numerous fundamental questions in systems neuroscience, including but not limited to (1) development of sensorimotor computation, (2) top-down mechanisms that control sensory navigation and whisker position, (3) cross-species comparison of active sensing. By comparing the sensorimotor exploration across wild-type juvenile and adult animals one could address how adaptive control of body and whisker position develop. Because adaptive motor control of whiskers is likely to be an outcome of a vector computation that ensures spatial constancy despite the coupled changes in the body [2], developmental changes in body positional control in respect to whisking might unravel the sequential

development of motor control. Repeating the same analysis across SERT KO, Fluoxetine and the corresponding control animals would help to address the role of serotonin in shaping motor development and consequences of altered serotonergic signaling in sensorimotor control in adulthood. Finally, by comparing the sensorimotor exploration between the multi-whiskered rats and mice one could address cross-species differences in adaptive motor control during object localization.

The data provided could serve the on-going machine learning efforts that will ultimately allow automated segmentation of whiskers in near real-time, i.e in temporal resolution shorter than the duration of a whisk cycle. To ensure the usability of the database as a training set, we have included ground-truth data from a subset of video recordings. Understanding the principles of active sensing in biological systems might help to instruct adaptive solutions for artificial systems to adapt sensory navigation to the ever-changing motor demands of the navigating agent.

Limitations

Freely behaving animal experiments are often burdened by high-dimensionality and the associated sampling limitations. Even if animals execute behavior in a constrained environment, e.g. exploring a stationary target while standing on an elevated platform, as in the behavioral experiments described herein, animals could change their approach angle, kinematics of whisking, duration of exploration, number of whisker used to sample the target, head angle and head elevation among other variables across different trials. Previous studies quantifying the sensory, motor and perceptual behavior during whisker based object localization showed that

both rats and mice perform spontaneous gap-crossing in a stereotypical manner and that ~100 trials (10 trials/animal) is sufficient to gather reproducible statistics of sensory and motor behaviors [1, 6, 8, 13, 15, 19]. Thus the current dataset with 6642 independent observations across 11 independent conditions (including species, age, genetic, pharmacological and sensory deprivation interventions) should provide sufficient sampling to address fundamental questions outlined in the previous section. However, we would like to attract the attention of the reader that the dataset does not include data from female animals.

Availability of the supporting data

Supporting data are available online (https://goo.gl/QhYe2X) and will be distributed via GigaScience DB.

List of abbreviations

SERT KO	Serotonin transporter knock-out rats, i.e. 5-HTT-/-
WT	Wild type controls accompanying the SERT KO, i.e. 5-HTT+/+
Fluox	Rats received Fluoxetine in drinking water
Vehicle	Control rats, drinking regular tap water

Competing interests

The authors declare no competing interests.

Author contributions

AlA compiled and organized the database. AlA, YZ, ArA, DvdW, MvdM performed data analysis and quality control. SM and LK performed data acquisition. SM performed pharmacological interventions. JH provided the transgenic rats. DS contributed to experimental design. RP supervised image processing. TC designed and supervised the project, wrote the first version of the manuscript. All authors edited otherwise approved the final version of the manuscript.

Bibliography

1. Celikel T, Sakmann B. Sensory integration across space and in time for decision making in the somatosensory system of rodents. Proc Natl Acad Sci USA. 2007;104:1395–400. doi:10.1073/pnas.0610267104.

2. Schroeder JB, Ritt JT. Selection of head and whisker coordination strategies during goal-oriented active touch. J Neurophysiol. 2016;115:1797–809. doi:10.1152/jn.00465.2015.

3. Haidarliu S, Kleinfeld D, Deschênes M, Ahissar E. The Musculature That Drives Active Touch by Vibrissae and Nose in Mice. Anat Rec (Hoboken). 2015;298:1347–58. doi:10.1002/ar.23102.

4. Huet LA, Hartmann MJZ. The search space of the rat during whisking behavior. J Exp Biol. 2014;217 Pt 18:3365–76. doi:10.1242/jeb.105338.

5. Arkley K, Grant RA, Mitchinson B, Prescott TJ. Strategy change in vibrissal active sensing during rat locomotion. Curr Biol. 2014;24:1507–12. doi:10.1016/j.cub.2014.05.036.

6. Voigts J, Herman DH, Celikel T. Tactile object localization by anticipatory whisker motion. J Neurophysiol. 2015;113:620–32. doi:10.1152/jn.00241.2014.

7. Clem RL, Celikel T, Barth AL. Ongoing in vivo experience triggers synaptic metaplasticity in the neocortex. Science. 2008;319:101–4. doi:10.1126/science.1143808.

8. Voigts J, Sakmann B, Celikel T. Unsupervised whisker tracking in unrestrained behaving animals. J Neurophysiol. 2008;100:504–15. doi:10.1152/jn.00012.2008.

9. Freudenberg F, Marx V, Seeburg PH, Sprengel R, Celikel T. Circuit mechanisms of GluA1-dependent spatial working memory. Hippocampus. 2013;23:1359–66. doi:10.1002/hipo.22184.

10. Celikel T, Marx V, Freudenberg F, Zivkovic A, Resnik E, Hasan MT, et al. Select overexpression of homer1a in dorsal hippocampus impairs spatial working memory. Front Neurosci. 2007;1:97–110. doi:10.3389/neuro.01.1.1.007.2007.

11. Mitchinson B, Martin CJ, Grant RA, Prescott TJ. Feedback control in active sensing: rat exploratory whisking is modulated by environmental contact. Proc Biol Sci. 2007;274:1035–41. doi:10.1098/rspb.2006.0347.

12. Grant RA, Sperber AL, Prescott TJ. The role of orienting in vibrissal touch sensing. Front Behav Neurosci. 2012;6:39. doi:10.3389/fnbeh.2012.00039.

13. Juczewski K, von Richthofen H, Bagni C, Celikel T, Fisone G, Krieger P. Somatosensory map expansion and altered processing of tactile inputs in a mouse model of fragile X syndrome. Neurobiol Dis. 2016;96:201–15. doi:10.1016/j.nbd.2016.09.007.

14. Hattox A, Li Y, Keller A. Serotonin regulates rhythmic whisking. Neuron. 2003;39:343–52.

15. Miceli S, Nadif Kasri N, Joosten J, Huang C, Kepser L, Proville R, et al. Reduced Inhibition within Layer IV of Sert Knockout Rat Barrel Cortex is Associated with Faster Sensory Integration. Cereb Cortex. 2017;27:933–49. doi:10.1093/cercor/bhx016.

16. Schubert D, Nadif Kasri N, Celikel T, Homberg J. Impact of monoaminergic neuromodulators on the development of sensorimotor circuits. In: Sensorimotor Integration in the Whisker System. Krieger P, Groh A, editors. Springer; 2015. p. 243–73.

17. Homberg JR, Olivier JDA, Smits BMG, Mul JD, Mudde J, Verheul M, et al. Characterization of the serotonin transporter knockout rat: a selective change in the functioning of the serotonergic system. Neuroscience. 2007;146:1662–76. doi:10.1016/j.neuroscience.2007.03.030.

18. Erzurumlu RS, Gaspar P. Development and critical period plasticity of the barrel cortex. Eur J Neurosci. 2012;35:1540–53. doi:10.1111/j.1460-9568.2012.08075.x.

19. Pang RD, Wang Z, Klosinski LP, Guo Y, Herman DH, Celikel T, et al. Mapping functional brain activation using [14C]-iodoantipyrine in male serotonin transporter knockout mice. PLoS ONE. 2011;6:e23869. doi:10.1371/journal.pone.0023869.

Click here to access/download
Supplementary Material
Responses to reviewers.pdf

Redlined manuscript

Click here to access/download
Supplementary Material
Azarfar_Redlined.pdf

Supplemental Table 1

Click here to access/download
Supplementary Material
Azarfar_Metadata_Supplemental Table1.xlsx

Cover letter Click here to download Personal Cover Azarfar_Cover letter.pdf

Donders Institute
for Brain, Cognition and Behaviour
Department of Neurophysiology

Professor Tansu Celikel, Ph.D.
Founding Chair, Department of Neurophysiology

Founding Director,
Dutch/German Graduate School in Bionics

Speaker, Donders Institute
Theme: Brain Networks and Neuronal Communication

Heyendaalseweg 135
6525 AJ, Nijmegen
the Netherlands

(+31) (0) 24 365 2702
www.ru.nl/neurophysiology
www.neurophysiology.nl
t.celikel@donders.ru.nl

August 30th, 2018

Dear Editor,

Please accept the attached manuscript, entitled "**An open-source high-speed infrared videography database to study the principles of active sensing in freely navigating rodents**", as a **Data Note** submission to Gigascience. This is a resubmission of our previously withdrawn manuscript, GIGA-D-16-00171.

Rodents are skillful navigators. Because they are nocturnal, and subterrenial in the wild, they predominantly use their tactile senses to navigate their environments. Over the last decade, the whisker system of rodents has become a popular model of active sensing. Our own work [1-5], for example, has shown that rodents employ frequency and amplitude modulation to actively control the position of their whiskers in space [1] as they integrate sensory information across whiskers in millisecond resolution [2]. Although rodents can perceive passive touch even before the onset of whisking [3], adaptive motor control is a close-loop computation [4] and requires mature sensory and motor processing, regulated (at least in part) by serotonergic signaling [5]. This sensorimotor computation enable rapid coordinate transformation as animals employ a form of Bayesian computation to control the position of their whiskers in the future based on the sensory information collected during the last ~90 ms [4]. Neural mechanisms of adaptive sensorimotor computation are some of the most fundamental problems in Neuroscience, and already helped to develop novel algorithmic solutions for adaptive navigation in artificial systems. Thus, **the field of active sensing continuously attracts a broad multidisciplinary audience**.

Here, we provide an extensive dataset that consists of 6642 high-speed videos (220-480 fps), recorded under infrared light as rats and mice located a stationary tactile target. Since our original submission, GIGA-D-16-00171, we have extensively processed data to match the requirements of the reviewers, added over 500 new videos where human observers manually segmented whiskers and nose of the navigating animals and re-written large portions of the manuscript. To ensure that the end-user of the data can take advantage of the dataset, we have developed two video processing software which are freely distributed via GitHub and references in the manuscript.

Our goal is to grow this database by systematically adding contextually classified new content; currently ongoing work include observation of navigation during object discrimination, home-cage navigation, exploration of novel-objects and novel spaces. We believe this undertaking will prove to be the prime resource for researchers across a broad range of disciplines, including cellular, behavioral, systems, cognitive neuroscience, and ethology, biomimetics, robotics, artificial intelligence, computer vision and active sensing communities, to study and model the principles of active sensing. Thus, we are encouraged to submit this Data Note submission for your consideration.

Thank you for your consideration in advance,

Sincerely yours,

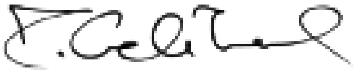

Tansu Celikel

References

1. Voigts et al. Journal of Neurophysiology,100:504
2. Celikel and Sakmann. Proceedings of the National Academy of Sciences, USA, 104:1395
3. Clem et al. Science, 319:101
4. Voigts et al. Journal of Neurophysiology,113:620
5. Miceli et al. Cerebral Cortex, 27:933
