# Supplementary material for: An open-source high-speed infrared videography database to study the principles of active sensing in freely navigating rodents
Source: Gigascience. 2018 Nov 10;7(12):giy134. doi: 10.1093/gigascience/giy134 (PMC6283211; doi:10.1093/gigascience/giy134)
Supplement: Response_to_Reviewer_Comments_Original_Submission.pdf [file giy134_response_to_reviewer_comments_original_submission.pdf]

Author's Response To Reviewer Comments

Close

We would like to thank the reviewers for their insight. The manuscript is now edited to fulfill the changes requested in the decision letter. We would like to thank the unmatched and continuous professional editorial support at Gigascience.

Close
